# Tropical cyclone FANTALA’s three turnbacks in the northeast of Madagascar Island

**DOI:** 10.1371/journal.pone.0305873

**Published:** 2024-08-30

**Authors:** Liqiong Zhang, Kequ Sun, Yulin Zhu, Yuhan Cao, Qi Feng, Yaoyao Zhou, Haibin LÜ

**Affiliations:** 1 School of Marine Technology and Geomatics, Jiangsu Ocean University, Lianyungang, Jiangsu province, China; 2 Lianyungang Meteorological Bureau, Lianyungang, Jiangsu province, China; 3 Nantong Marine Center of Ministry of Natural Resources, Nantong, Jiangsu province, China; Guangdong Ocean University, CHINA

## Abstract

The unique Tropical cyclone (TC) Fantala appeared in the central Indian Ocean (12.4°S, 73.5°E) at 00Z on April 11 in 2016 and moved northwestward along the northeast of Madagascar at 18 Z on April 15. Then, two incomprehensible turnbacks formed a unique TC track. The dynamic mechanisms of the three turnbacks were first studied based on remote sensing and multisource reanalysis data. The results reveal that the wind field with upper divergence and lower convergence promotes the development of Fantala. The anticyclone high pressure on the middle level atmosphere is an important factor for TC turnbacks. On 15 April, the TC made the first turnback to turn northwest due to the southward anticyclone weakened to moving northwest. On 18 April, the TC made the second turnback along the anticyclone edge due to the northern high-pressure and southern low-pressure trough. On 22 April, the TC made the third turnback because the anticyclonic high press center broke into two small independent anticyclonic centers in the southwest and northeast, which created a barrier band and pushed the northern TC to move to the northwest. Meanwhile, the vertical wind shear (VWS) also provides favorable conditions for TC turnbacks. On April 18, the middle atmosphere of the TC was affected by strong easterly shear and weak southerly shear, and the second turnback was completed. On April 22, the middle level environment was affected by strong westerly shear and weak north shear, and the third turnback was completed. Additionally, heat transport from the ocean to the atmosphere provides favorable conditions for TC development. On April 18, The maximum mean latent heat flux over northeastern Madagascar was 112.94 W/m^2^, Tropical Cyclone Heat Potential was 39.05 kJ/cm^2^, and the maximum wind speed at the center of the TC was 155 kts. On April 22, The heat transfer from the equator increased by 18.08 W/m^2^ compared with the latent heat on 21 April, the Tropical Cyclone Heat Potential was 33.30 kJ/cm^2^, the maximum wind speed in the TC center was 90 kts, the high PV centerspread down from 850 mb to 900 mb. This study deepens the understanding of track forecasting during the development of a TC.

## Introduction

A tropical cyclone (TC) is a powerful cyclonic vortex with a warm central structure that usually occurs over tropical and subtropical oceans. When a TC develops into a tropical storm (TS), strong winds, heavy precipitation and storm surges usually occur, causing serious damage to the affected regions [1]. Therefore, it is necessary to analyze the interaction between ocean and atmosphere, which is important for predicting the track of TCs and strength. The northeastern Madagascar Basin is one of the tropical basins most prone to TCs [2], with the strongest TC in January and the highest TC frequency from December to March. The occurrence of TCs often leads to disastrous environmental and socioeconomic impacts [3, 4]. Additionally, internal waves have also been reported during and after the passage of TCs, which have important effects on ocean platforms [5]. Strong TCs can cause turbulent mixing, which can cause the sediment to resuspend and provide favorable conditions for Chl-a blooms. The strong air-sea interactions caused by TCs can have an important impact on the marine engineering environment. The method of establishing instantaneous water level model for tidal correction has been studied [6]. Convolutional neural network [7]and function model [8] were used to interpret the remote sensing image accurately. Multi-sensor data fusion method [7] was used to detect buried pipelines in the seabed to protect marine engineering environment and marine biodiversity.

**Fig 1:**
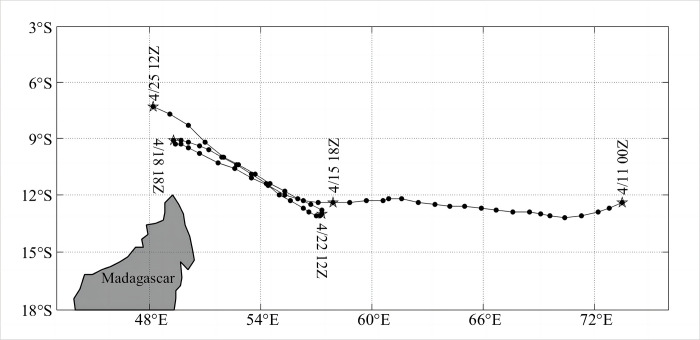
Path of TS Fantala. The positions in the center of the TC are marked with black circles with the time format of month-date-hour. The pentagram represents the location of the TC center primary changes in the path.

The formation mechanism of TCs is quite complicated. Gray [9] suggested that the following requirements must be met for the formation of a TC: a low-level vortex, a sufficiently large Coriolis force, minimal vertical wind shear in the upper and lower troposphere, a sea temperature exceeding 26°C, atmospheric instability, and high humidity in the mid-troposphere. The strength of a TC can be influenced by a number of complicated physical processes, involving TC internal kinetics [10] atmospheric flow fields, and interactions between ocean and atmosphere. Interactions between ocean and atmosphere play an essential role in the formation of TCs [11]. TCs can be the result of the continuous absorption of ocean energy and water vapor by latent and sensible heat exchange at the air-sea surface. The atmospheric pressure field can have an impact on the track and intensity of TCs, and the change in the direction and intensity of TCs will also have an impact on the high-pressure field [12]. Wind shear at different heights has different effects on the development of the vertical motion of TCs. If the difference between the upper and lower wind directions is within the threshold range, then the upper and lower typhoon cloud systems are stably concentrated in the same center. The upper divergence of wind will promote the developed vertical motion for TCs. The TC Fantala appeared in the central Indian Ocean (12.4°S, 73.5°E) at 00Z on April 11 in 2016 and made the first northwestward turnback along the northeast of Madagascar at 18Z on April 15 (Fig 1). It is worth noting that *Fantala* made the second southeastward reverse turnback at 18Z on April 18 and the third northwestward reverse turnback at 12Z on April 22.

This paper mainly reveals the dynamic mechanism of the TC’s three turnbacks. The data and methods, the results and discussion, the conclusions are presented in order below.

## Data and method

### Data

The track of TS Fantala was acquired from the Joint Typhoon Warning Center (JTWC) (obtainable at https://www.metoc.navy.mil/jtwc/jtwc.html). TS data included the time, wind speed and central location at 6-hour intervals. The moving speed of a TS can be measured in accordance with the positions of the TS center.

Atmospheric reanalysis data, including geopotential heights, wind field and potential vorticity (PV), were mainly acquired from the hourly ERA5 dataset of the European Centre for Medium-Range Weather Forecasts (ECMWF)(obtainable at https://www.ecmwf.int/) with a spatial resolution of 0.25°×0.25°.

SST with a spatial resolution set to 0.25° was optimized for interpolation with an everyday measurement product provided by Remote Sensing Systems (http://www.remss.com/).

Latent heat fluxes were from the NOAA Climate Data Record (CDR) of Ocean Heat Fluxes, version 2 (https://www.ncei.noaa.gov/) with a horizontal resolutions of 0.25°.

Horizontal wind data were obtained from NCEP/NCAR (National Center for Environmental Prediction/National Center for Atmospheric Research) reanalysis data (available at https://www.psl.noaa.gov/).

## Methods

### Ekman pumping velocity (EPV)

The Ekman pumping velocity (EPV) can lead to the convergence and divergence of upper seawater [13], which can be expressed as follows:

$$EPV=\mathrm{curl}\left(\frac{\Delta \times \tau }{\rho_{f}}\right)$$ (1)

$$\tau =\rho_{a}C_{d}u^{2}$$ (2)

f=2Ωsinθ (3)

$$C_{d}=1.2\times 10^{‐3},u<11m\cdot s^{‐1}$$

$$=(0.49+0.065u)\times 10^{‐3},11<u<25m\cdot s^{‐1}$$ (4)

$$=2.115\times 10^{‐3},u>25m\cdot s^{‐1}$$

where Δ×τ is the curl of surface wind stress, ρ is the density of seawater, *f* is the Coriolis parameter, Ω is the rotational angular velocity of the Earth, θ is the latitude, τ is wind stress, ρ_α_ is the density of atmosphere, C_d_ is the drag coefficient of wind and u is the wind velocity at 10 meters above sea level.

### Vertical wind shear (VWS)

The shear profile of VWS often has an important effect on the TC development. The deep-level VWS is usually calculated with the difference in wind velocity between 850 hPa and 200 hPa according to Eqs (5)–(7): [14, 15]

$${\bar{\Delta u}}_{200-850}=\frac{\sum_{1}^{n}\left(u_{200i}-u_{850i}\right)}{n}$$ (5)

$${\bar{\Delta v}}_{200-850}=\frac{\sum_{1}^{n}\left(v_{200i}-v_{850i}\right)}{n}$$ (6)

$$VWS=({\bar{\Delta u}}_{200-850})\cdot \overset{\to }{l}+({\bar{\Delta v}}_{200-850})\cdot \overset{\to }{J}$$ (7)

where u_200_ (u_850_) is the zonal wind speed at 200 hPa (850 hPa). v_200_ (v_850_) is the meridional wind speed at 200 hPa (850 hPa). The low-level VWS is usually from the difference in wind velocity between 700 hPa and 925 hPa, which can be obtained with the same method as that for the deep-level VWS [16]. $\overset{\to }{l}$ represents the direction of the u, with positive values east and negative values west, $\overset{\to }{J}$ represents the direction of the v, with positive values north and negative values south. n is the sample size.

### Tropical Cyclone Heat Potential

The ocean heat content (OHC) of the upper ocean can be better represented by the 26°C isotherm depth (D_26_), which known as the Tropical Cyclone Heat Potential can be used to describe the heat loss of the upper ocean [17] using Eq (8).

$$TCHP=\rho C_{\rho }\int_{0}^{D_{26}}(T-26)dZ$$ (8)

where ρ = 1024kg/m^3^ is the seawater density, C_ρ_ = 4.0×10^3^J·kg^-1^·°C^-1^ is the specific heat capacity of seawater, T is the seawater temperature at dz, and D_26_ is the depth of 26°C isotherm.

## Results and discussion

### Atmospheric environment

#### Wind field and geopotential height

**Fig 2:**
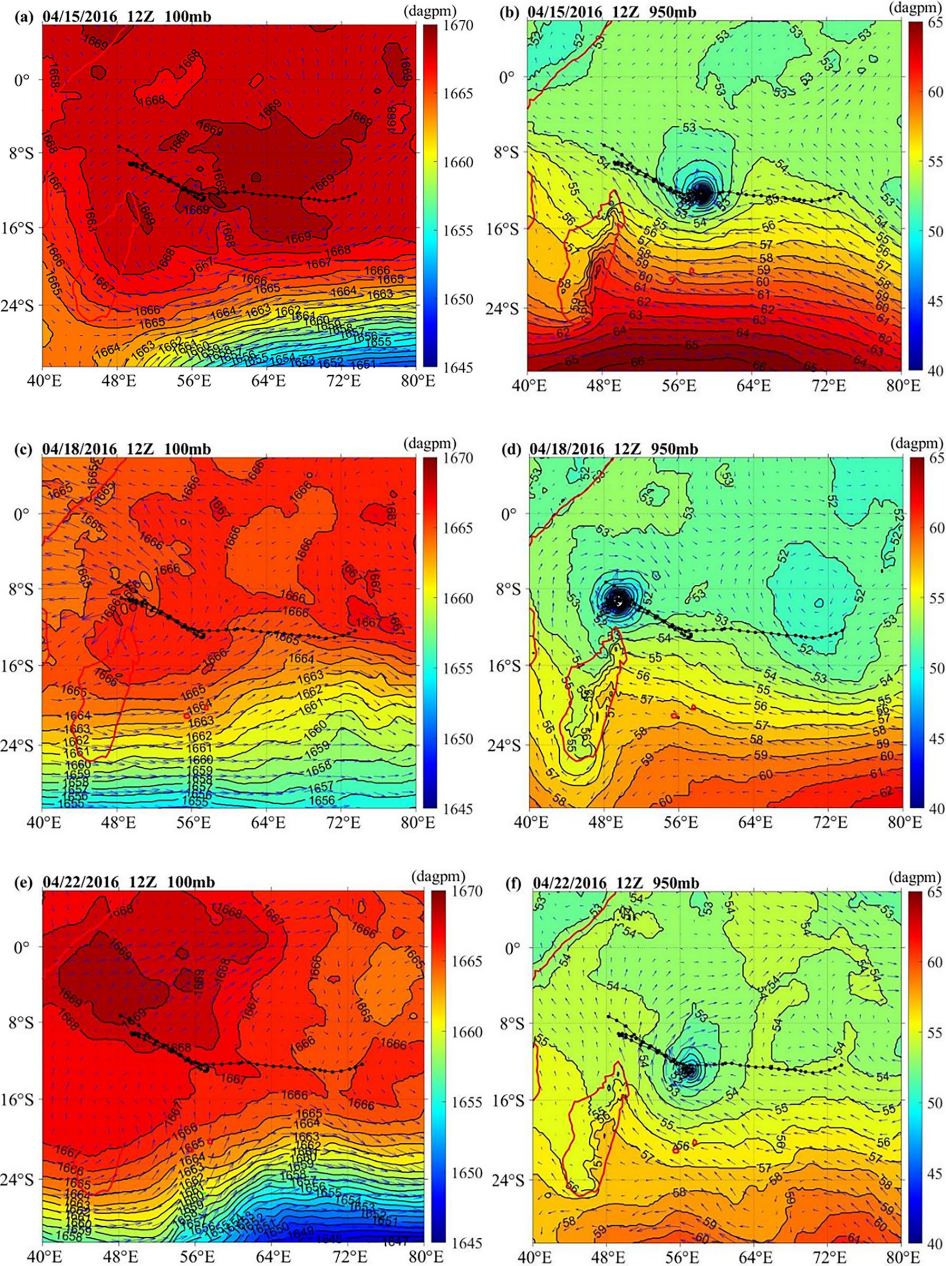
The geopotential height and wind field distribution at 100 mb and 950mb on 12Z on April 15, April 18 and April 22, 2016. Color bars represent geopotential heights (unit: dagpm). (a), (c) and (e) show atmospheric pressure at 100 mb, and (b), (d) and (f) show atmospheric pressure at 950 mb.

Fig 2 provides the geopotential height and wind field distribution of TC Fantala at 12Z on April 15, 12Z on April 18 and 12Z on April 22, 2016. The influence of the high-altitude system on TC could be noticed at 100 mb (Fig 2A, 2C and 2E); the upper level over the northeastern Madagascar Sea area was controlled by a high pressure system surrounded by the 1667 dagpm contour line, which benefited from the upper atmospheric divergence (Fig 2A). The major high-pressure section forms an anticyclonic circulation with a center greater than 1669 dagpm, which was situated on Fantala’s track at 12Z on April 22 (Fig 2E). Stable upper divergence conditions were provided at the upper-air system for TC reinforcement. TC Fantala is a low-pressure vortex that can be clearly observed at 950 mb (Fig 2B, 2D and 2F). Northeastern Madagascar was mainly dominated by a low pressure, with a distinct low pressure trough surrounded by the 54 dagpm contour (Fig 2B). The geopotential height in the low-pressure center was less than 42 dagpm at 12Z on April 18 (Fig 2F), which benefited from the lower convergence. Therefore, the wind field with upper atmospheric divergence and lower convergence helps to the TC development. The similar occurrence are also found in Atlantic TC genesis events [18] and the Bay of Bengal sea area [11].

**Fig 3:**
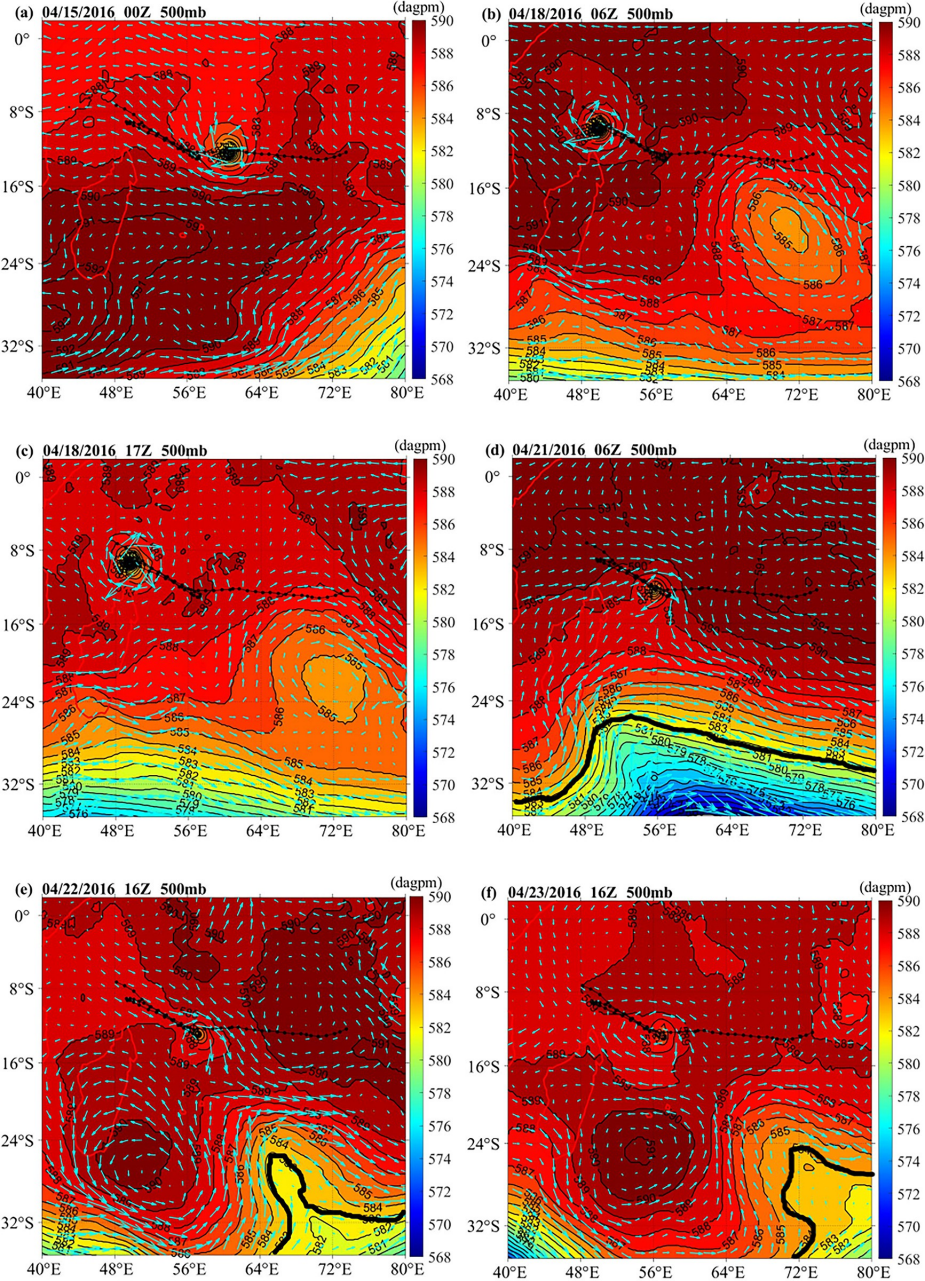
The geopotential height and wind field distribution at 500 mb on (a) 15 April 2016 00:00, (b) 18 April 2016 06:00, (c) 18 April 2016 17:00, (d) 21 April 2016 06:00, (e) 22 April 2016 16:00, and (f) 23 April 2016 16:00.

The distributions of the geopotential height and wind field at 500 mb are shown in Fig 3. On April 15, the high-pressure system of 16°S-35°S was located south of the TC path (Fig 3A). Because the high-pressure system moved to the northwest, the TC turned to the northwest along the edge of the 589 dagpm high-pressure field on April 15 (Fig 3A), and the first turback happened. On April 18, the high pressure was located in the northwest (6°N-23°S, 30°E-60°E) (Fig 3B). Meanwhile, the low-pressure trough moved northward when the TC suddenly moved backward to the southwest (Fig 3C). Due to the northern high-pressure and southern low-pressure trough, the TC continued to move southeastward (Fig 3D), and the second reverse turnback appeared [19]. On April 22, TC Fantala made its third reverse turnback because the anticyclonic high press center broke into two small independent anticyclonic centers in the southwest and northeast, which created a barrier band and pushed the TC to move to the northwest (Fig 3E and 3F) [10].Similar to previous studies, The 500 mb geopotential height play a crucial role in the intensification of the unique storm Deliwe (2014) in the Mozambique Channel [20].

### VWS

**Fig 4:**
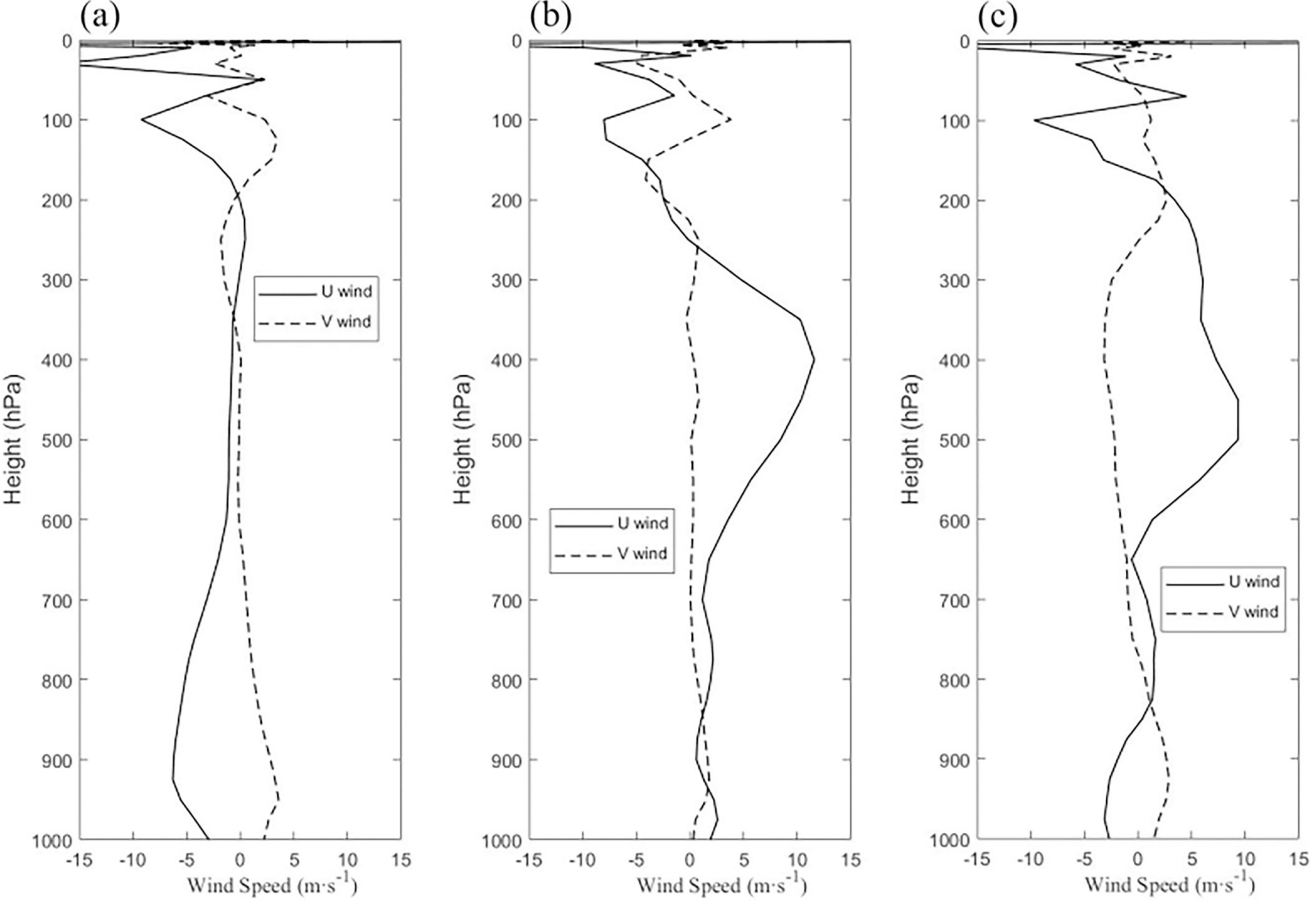
Variation in mean meridional (u_wind) (m/s) and zonal (v_wind) (m/s) winds at (a) 00:00, 18 April, (b) 12:00, 18 April, and (c) 23:00, 18 April 2016.

**Fig 5:**
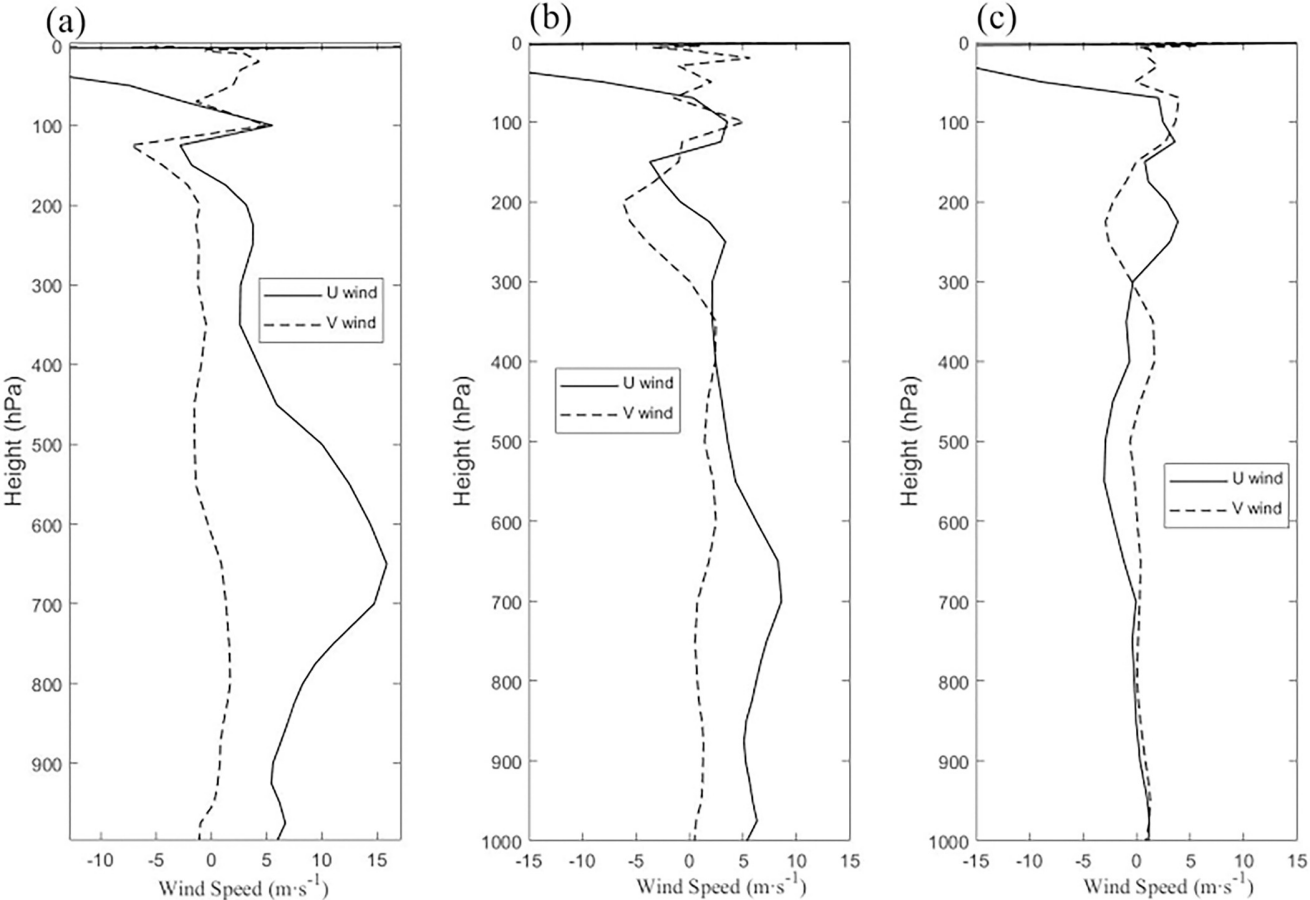
Variation in mean meridional (u_wind) (m/s) and zonal (v_wind) (m/s) winds at (a) 16:00, 21 April, (b) 22:00, 21 April, and (c) 03:00, 22 April 2016.

The relationship between the VWS and vertical wind has been paid more attention by some scholars. Corbosiero and Molinari [21] introduced three mechanisms for vertical movement on the left side of downshear. Nevertheless, Finocchio found that VWS occurred at different locations under separate conditions in the tropical northern hemisphere, where both high and low levels could be influenced by VWS. Figs 4 and 5 show the changes in the mean meridional and zonal winds at different altitudes before and after the last two TC reverse turnbacks.

**Fig 6:**
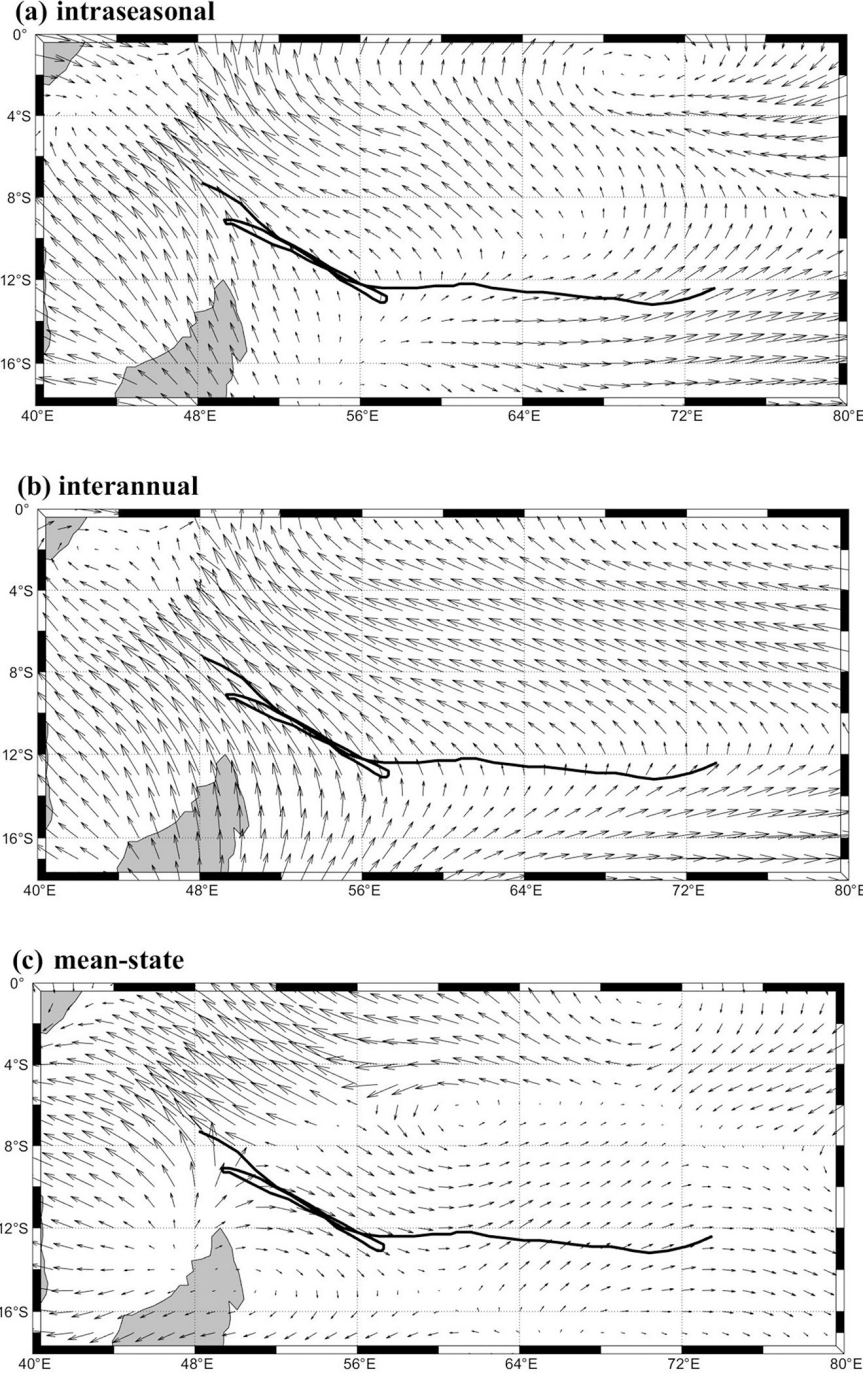
Composites of the vertically averaged (850–300 hPa). (a) intraseasonal, (b) interannual, and (c) mean-state horizontal winds (m⋅s^−1^) from 00:00 UTC on Apr.11 to 18:00 UTC on Apr.25.

Before and after the last two turnbacks, the meridional and zonal winds at the TC center ± 2° latitude for the 24 h were shown in Figs 4 and 5. At 06:00 on Dec. 9, the shear mainly happened below 600 hPa (Fig 5A). At 12:00 on Apr. 18, the shear still mainly appeared at the middle levels between 400 hPa and 600 hPa and high levels above 100 hPa, which changes from a westward direction at 200 hPa to an eastward direction at high levels above 100 hPa. TC intensity became the highest from 00:00 on Apr. 18 (Table 1). In Fig 6B, the shear is observed mainly at high levels above 200 hPa, which transforms an eastward direction at 700 hPa to a westward direction at 600 hPa. In Fig 6C, it can be found that the shear changes from south to north at the middle level between 300 and 700 hPa on Apr. 22. The VWS above 200 hPa was always existent, which was consistent with the VWS in Figs 4 and 5. The VWS changed from a westward direction at 200 hPa to an eastward direction at high levels above 100 hPa. These dynamics were consistent with the results of Xia, et al., (2023) who found that the vertical wind shear of TC Madi (2013) was enhanced below 700 hPa and influenced Madi’s turn to the southwest on 10 December [22]. The deep-level VWS has an integral influence on TC movement, which is better organized by middle-level shear [23].

**Table 1:** Characteristic parameter information of TS Fantala.

Date	Lat (°S)	Lon (°E)	MWS(m·s^-1^)	MLSP^b^ (mb)
4/11/2016 00Z	12.4S	73.5E	10.28	1007
4/11/2016 18Z	13.1S	71.3E	20.57	993
4/12/2016 18Z	12.9S	68.5E	33.43	974
4/13/2016 18Z	12.6S	65.0E	46.29	956
4/14/2016 18Z	12.2S	61.6E	51.44	948
4/15/2016 18Z	12.4S	57.9E	66.87	926
4/16/2016 18Z	11.5S	54.4E	69.44	922
4/17/2016 06Z	10.6S	52.6E	74.58	922
4/18/2016 00Z	9.5S	50.1E	79.73	907
4/19/2016 12Z	9.4S	50.7E	43.72	952
4/20/2016 12Z	10.9S	53.5E	54.01	944
4/21/2016 12Z	12.7S	56.3E	41.15	963
4/22/2016 00Z	13.1S	57.0E	46.29	956
4/23/2016 12Z	11.8S	55.3E	18.00	989
4/24/2016 18Z	9.2S	51.0E	12.86	1006
4/25/2016 12Z	7.3S	48.2E	12.86	1007

Note: Times are given in UTC (denoted as Z).

MWS: Maximum wind speed. MLSP^b^: Minimum sea level pressure

### Environmental flows with different timescales

The averaged composites of horizontal winds from 850 hPa to 300 hPa can be used to reveal the northward movement of Typhoon Sanba [24],which contains intraseasonal, interannual and mean-state horizontal winds. Here, composites of the vertically averaged winds from 850 to 300 hPa are shown in Fig 6. Fantala was generated at 12.4°S, 73.5°E. With the anticyclonic atmospheric circulation system (Fig 6A and 6C), the northerly mean airflow and intraseasonal southeasterly trade winds contribute to the northwestward movement of Fantala between 4°S and 16°S. However, the mean-state horizontal airflow caused Fantala to move southeastward (Fig 6C). The southeast trade winds are stronger than the interannual winds at 12°S and the average airflow is southward in the east of 48°E, where the complex wind direction also influences the formation of the three turnbacks of TC fantala.

### Atmospheric potential vorticity

**Fig 7:**
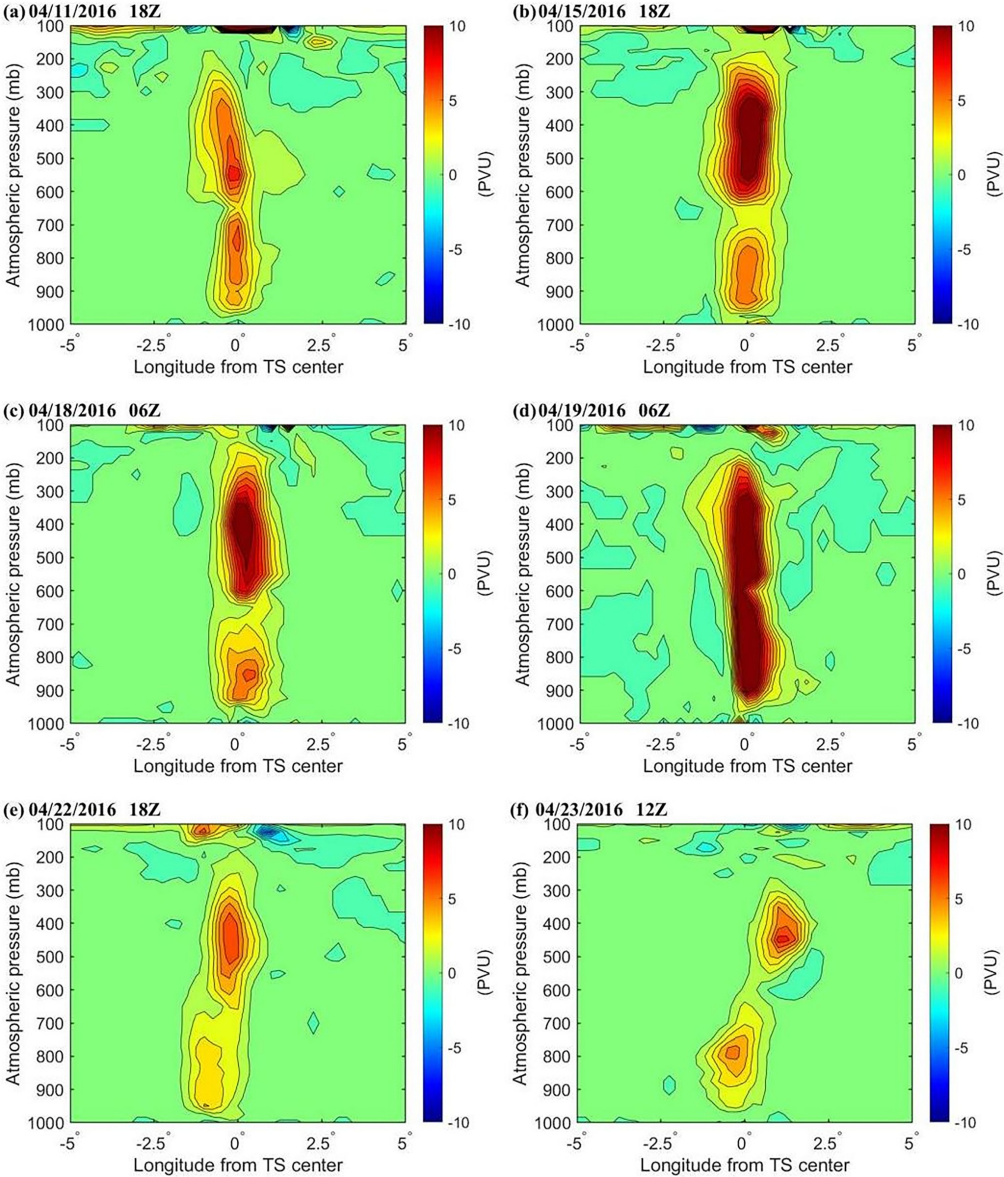
The change in the zonal vertical cross-section of the PV of ± 5° longitude over the TC center during TC development (unit: PVU, 1PVU = 106 K m^2^ kg^-1^ s^-1^).

**Fig 8:**
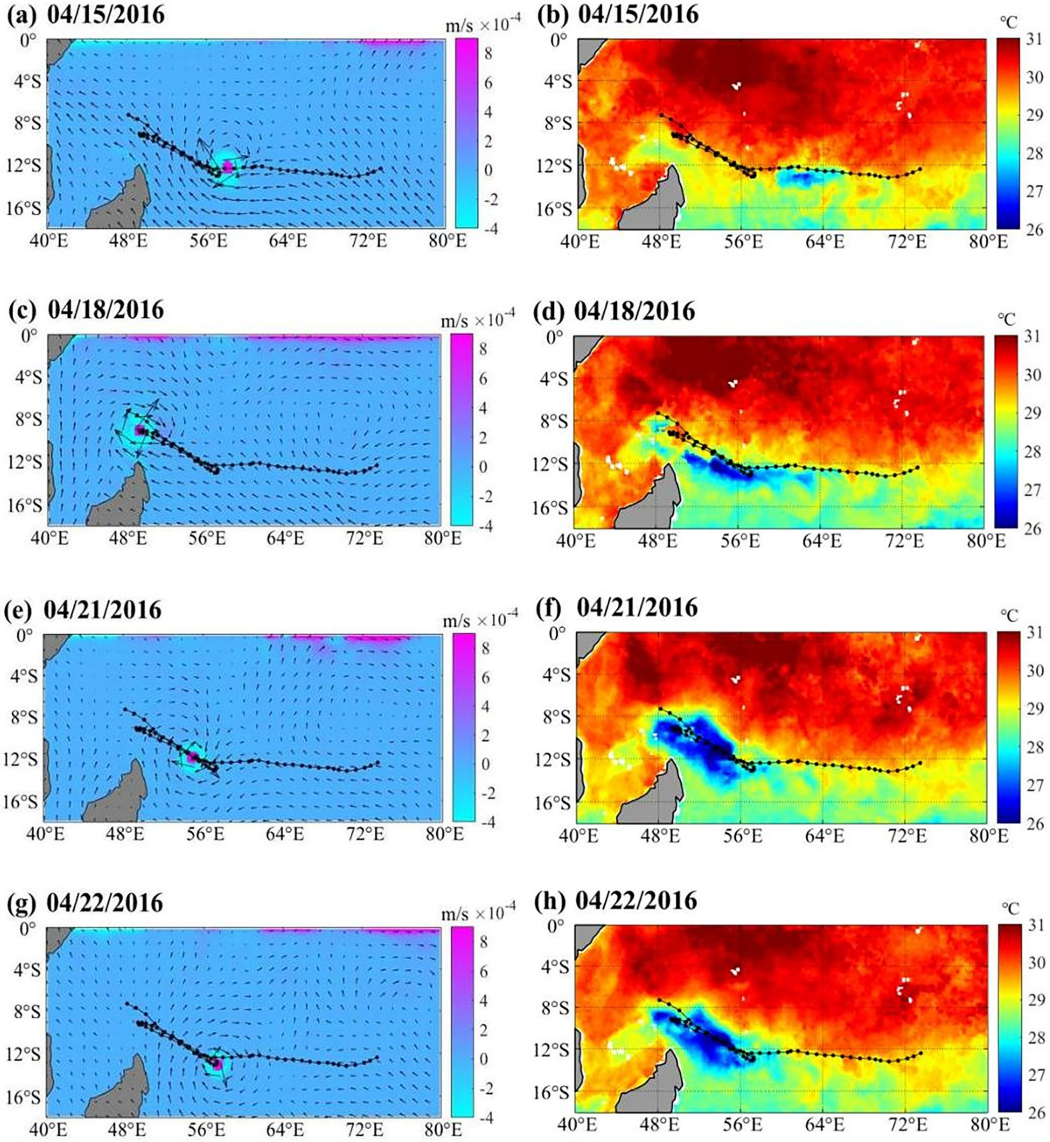
Marine environmental fields during the passage of the TC. (a), (c), (e), and (g) show the changes in the EPV (color bar) and wind fields (arrow) on April 15, April 18, April 21 and April 22, 2016, respectively. Positive (negative) values indicate upwelling (downwelling). (b), (d), (f), and (h) show the changes in SST (color bar) on April 15, April 18, April 21 and April 22, 2016, respectively.

**Fig 9:**
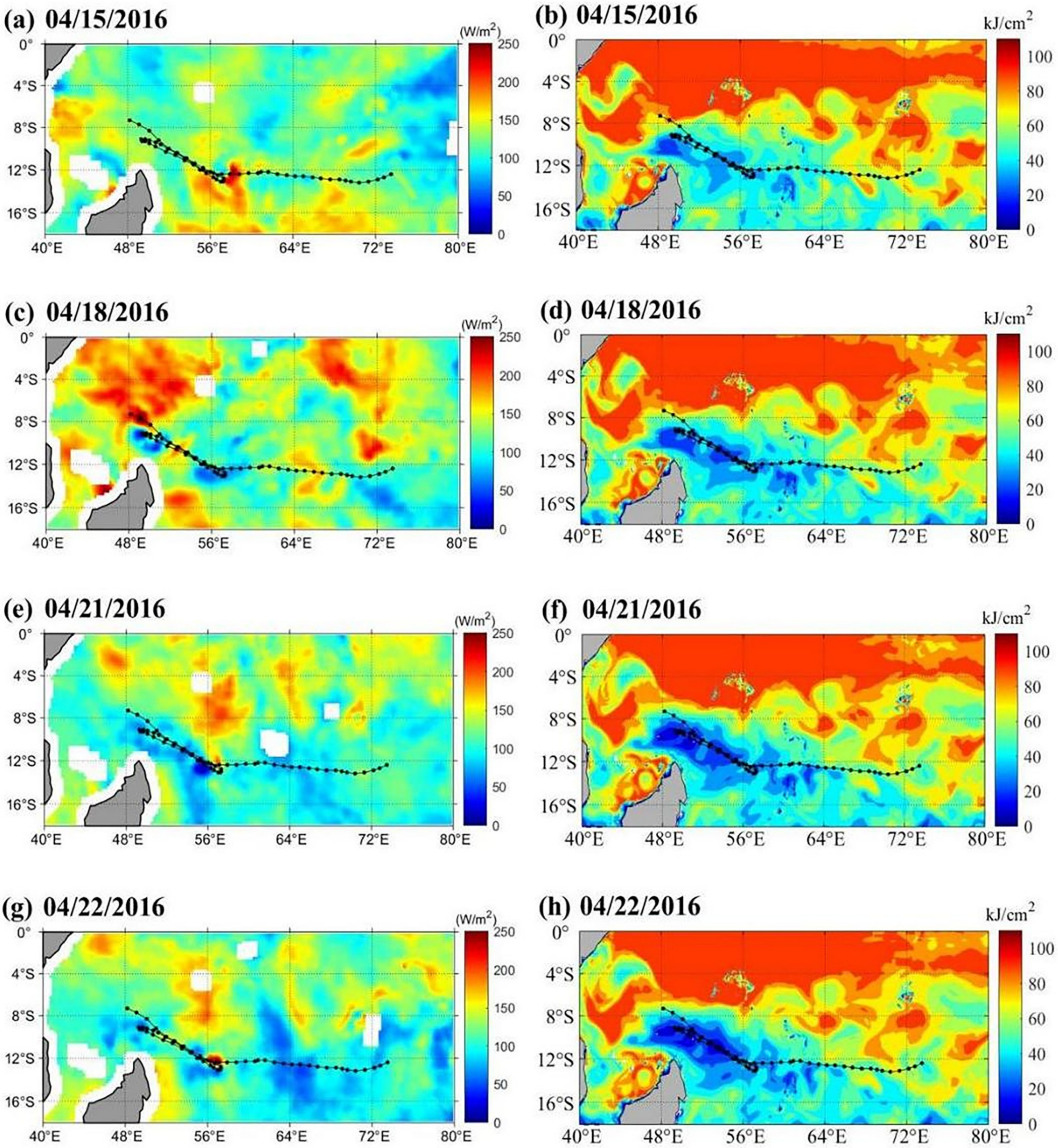
Heat changes in the ocean during the passage of the TC. (a),(c),(e),(g) show latent heat flux on April 15, April 18, April 21 and April 22, 2016, respectively; (b),(d),(f),(h) show Tropical Cyclone Heat Potential on April 15, April 18, April 21 and April 22, 2016, respectively.

Potential vorticity (PV) has an important effects on the internal structure and strength of a TC. As the cyclone rotation accelerates, the air column is elongated with PV increases. In contrast, when the air column rotation slows, it is shortened with PV decreases. Fig 7 presents the variations in the zonal vertical cross-section of the PV of ± 5° longitude over the TC center. On April 15, large amounts of latent heat were extracted from the sea surface (Fig 8A), with a high PV center at heights of 450 mb and 850 mb (Fig 7B). The maximum PV reached 11.8 PVU at a height of 450 mb and 5.742 PVU at a height of 850 mb. These high PV zones are clearly independent of the air above the stratosphere and are likely the product of latent heat released from the tropical ocean (Figs 7B and 8D). On April 18, there were high PV centers at heights of 400 mb and 850 mb, and the PV at a height of 400 mb was stronger (Fig 7C). Meanwhile, the positive vorticity column extends to the tropopause, and the middle layer high PV propagates downward, resulting in TC development in the lower layer (Fig 7D), and the second TC turnback appeared. On 22 April, due to the heat transfer from the equator, the increase in latent heat on 21 April was 18.08 W/m^2^, and the TC was slightly stronger (Fig 9). The high PV center at a height of 850 mb spreads down to 900 mb, the PV center range increases, and the third TC turnback occurs. On 23 April, the central air column of the TC shortened and diverged, the PV decreased, and the TC intensity weakened significantly (Fig 7F).

In Bell and Montgomery’s (2008) study of category 5 hurricane Isabel, the PV was found to have a maximum value at 3 km from the hurricane center. These PV dynamics are consistent with our findings [25].

### Marine environment

#### SST and EPV

SST is one of the factors affecting the moving path of TCs. The intensity and trajectory of a TC is largely determined by the energy exchange between the ocean and atmosphere. TCs can extract energy from the warm ocean surface to maintain or even increase their intensity [26]. In Fig 8, the left figures show the changes in EPV during the development of the TC from 15 April to 22 April, and the right figures show the changes in SST during the development of the TC. Fig 8B shows that the SST in a large area of northeastern Madagascar is higher than 29°C, where the TC occurs. During the development of the TC, the SST in the northeastern Madagascar decreased significantly (Fig 8D, 8F and 8H). The upwelling at the TC center is stronger, where the maximum EPV reaches 8.86×10^−4^ m/s on April 18 (Fig 8C). During the passage of the TC, obvious sea surface cooling with the upwelling appeared over the study area (Fig 8D, 8F and 8H), and weakened the heat supply of the TC after April 18.

#### Heat change by upper ocean

**Fig 10:**
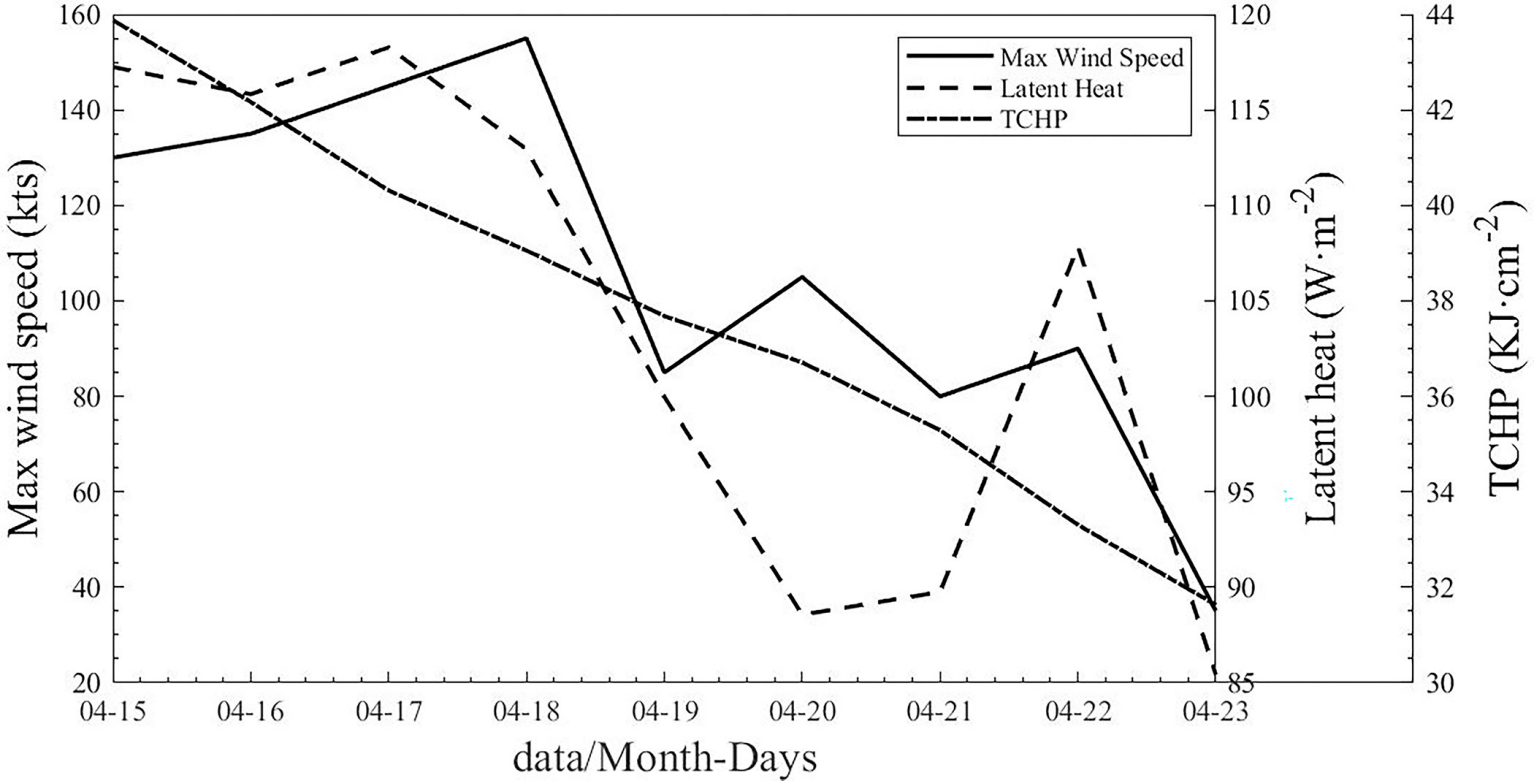
Mean maximum wind speed, latent heat flux and Tropical Cyclone Heat Potential from 15 to 23 April.

During the development of the TC, obvious thermal changes appeared in the upper ocean (Figs 9 and 10). On April 15 (April 17), the maximum latent heat flux over the TC was 117.24 W/m^2^(118.27 W/m^2^). The latent heat flux decreased significantly after April 18. The maximum latent heat flux over the sea area of the TC center was 88.57 W/m^2^ on April 20 and 89.75 W/m^2^ on April 21 (Figs 7B–7F, 9 and 10). After the passage of a TC, the upper oceans lose energy, and their accumulated energy is transferred to the TC. The heat content in the upper seawater before the TC passed was significantly higher than that after the TC passed [11]. Tropical Cyclone Heat Potential decreased from 43.88 kJ/cm^2^ on April 15 to 31.62 kJ/cm^2^ on April 23, a loss of 12.25 kJ/cm^2^. At the same time, the TC also stimulated sharp cooling of the sea surface, and the Tropical Cyclone Heat Potential in the upper ocean decreased significantly (Fig 10). The three turnbacks were accompanied by the response of the upper ocean, such as the decrease in latent heat of the sea surface (Fig 9G), SST (Fig 8G), Tropical Cyclone Heat Potential (Fig 9H), and significant heat loss of the upper ocean (Fig 10) [27]. This phenomenon has also appeared in the western pacific, when there were important effects of a warm oceanic feature on the super typhoon Hinnamnor in August 2022 [28].

## Conclusions

TC Fantala appeared in the middle of the South Equatorial Current in the central Indian Ocean on April 11, 2016. Three turnbacks occurred on the TC track during the passage of Fantala. The dynamic mechanisms of the three turnbacks were studied based on remote sensing and multisource reanalysis data. The following conclusions can be drawn:

1. The wind field with upper atmospheric divergence and lower convergence provides a favorable environment for the development of Fantala. The anticyclone high pressure on the middle level atmosphere is an important factor for TC turnbacks. On 15 April, the TC made the first turnback northwestward due to the southward anticyclone moving northwest. On 18 April, Fantala made its second turnback along the anticyclone edge at 590 dagpm due to the northern high pressure and southern low pressure trough. On 22 April, Fantala made its third turnback because the anticyclonic high press center broke into two small independent anticyclonic centers in the southwest and northeast, which created a barrier band and pushed the TC to move to the northwest.
2. The VWS provides ideal conditions for TC turnbacks. On April 18, the middle atmosphere of the TC was affected by strong easterly shear and weak southerly shear, and the second reverse turnback was completed. On April 22, the middle level environment was affected by strong westerly shear and weak north shear, and the third reverse turnback was completed. The southeast trade winds are stronger than the interannual winds at around 12°S and the average airflow is southward in the east of 48°E, where the contrasting wind direction help to the formation of the turnbacks of TS fantala.
3. Heat transport from the ocean to the atmosphere provides favorable conditions for TC development. On 18 April, the maximum mean latent heat flux over northeastern Madagascar was 112.94 W/m^2^, Tropical Cyclone Heat Potential was 39.05 kJ/cm^2^, and the maximum wind speed at the center of the TC was 155 kts. The positive vorticity column elongates and extends to the tropopause, which is supported by the upper atmosphere and latent heat flux when the second reverse turnback occurs. On 22 April, the heat transfer from the equator increased by 18.08 W/m^2^ compared with the latent heat on 21 April, the Tropical Cyclone Heat Potential was 33.30 kJ/cm^2^, the maximum wind speed in the TC center was 90 kts, the high PV centerspread down from 850 mb to 900 mb.
